# Twelve-month Results From the Percutaneous Endoscopic Benign Biliary Laser Stricturotomy Study: A Prospective Single-Arm Pilot Trial Evaluating Safety and Efficacy

**DOI:** 10.1016/j.gastha.2025.100770

**Published:** 2025-08-25

**Authors:** Dustin G. Roberts, Abinaya Ramakrishnan, Aniket Joglekar, James Sayre, Zachary Haber, Ravi Srinivasa

**Affiliations:** 1 Department of Radiology, Division of Interventional Radiology, Ronald Reagan UCLA Medical Center, Los Angeles, California; 2 David Geffen School of Medicine, University of California, Los Angeles, California

**Keywords:** Benign biliary stricture, Ho:YAG laser, holmium laser, interventional radiology, laser stricturotomy, percutaneous cholangioscopy

## Abstract

**Background and Aims:**

Benign biliary strictures (BBSs) are associated with poor long-term patency, substantial morbidity, and high health-care costs. Despite a multitude of available treatment options—peroral endoscopic, percutaneous transhepatic, and surgical reconstruction—recurrence rates remain high. This study assesses the safety and efficacy of percutaneous transhepatic cholangioscopy (PTCS)–assisted holmium laser stricturotomy for BBS management.

**Methods:**

The Percutaneous Endoscopic Benign Biliary Laser study is a prospective, single-arm, open-label pilot trial conducted at a single academic institution. Between October 2022 and August 2023, 5 patients with BBSs underwent PTCS-assisted laser stricturotomy, with or without lithotripsy, using a holmium:yttrium-aluminum-garnet laser fiber. Patients were followed for 12 months. Primary endpoints included technical success and 30-day adverse events, while secondary endpoints assessed 12-month primary patency (freedom from stricture recurrence) and cumulative device-free survival. Data were gathered through imaging (magnetic resonance cholangiopancreatography or CT), clinical visits, and laboratory tests at 3, 6, and 12 months.

**Results:**

Technical success was achieved in 100% of cases, with all patients experiencing stricture resolution and biliary drain removal. Primary biliary patency was 80% (4 of 5), with 1 patient experiencing asymptomatic stricture recurrence and undergoing metallic stenting, remaining drain-free thereafter. Cumulative device-free survival averaged 9.4 ± 4.8 months. Three 30-day adverse events were noted in 2 patients: self-limited hemobilia, a bile leak, and a tract site infection.

**Conclusion:**

PTCS-assisted holmium laser stricturotomy is a promising new treatment for benign biliary strictures, offering durable tube-free 12-month patency with an acceptable safety profile. The Percutaneous Endoscopic Benign Biliary Laser study provides a foundation for future clinical research to build upon and suggests that this emerging approach could augment traditional, less effective stenting protocols.

## Introduction

The benign biliary stricture (BBS) poses a significant treatment challenge that continues to elude endoscopists and interventional radiologists alike. Despite its benignity, the BBS is notorious for its association with devastating patient morbidity, poor quality of life, low long-term patency, and high health-care costs and resource utilization.^1^, ^2^, ^3^ Over 80% of BBS are iatrogenic—the majority due to complications from cholecystectomy and orthotopic liver transplantation.^1^, ^2^, ^3^, ^4^, ^5^ Impediment to normal antegrade bilioenteric flow causes a state of chronic cholestasis that becomes a catalyst for calculi, recurrent cholangitis, and life-threatening sepsis.^6^ Patients often suffer from chronic bouts of biliary colic and jaundice, and if left untreated, can eventually develop biliary cirrhosis.

Broadly, there are 3 approaches to treating BBS: peroral endoscopic (retrograde approach), percutaneous transhepatic (antegrade approach), and surgical biliary reconstruction.^7^ Most contemporary clinical guidelines recommend a peroral-first approach with endoscopic retrograde cholangiopancreatography (ERCP); however, patients with altered anatomy or particularly challenging strictures often require percutaneous access by interventional radiology (IR).^8^ Unfortunately, current standard practices involve prolonged stenting protocols and frequent interventions to gradually dilate the stricture over time, yet long-term stricture recurrence rates approach 30%–50% regardless of the technique,^5^ leaving many patients reliant on lifelong drainage catheters or stents. Hence, the prevailing goal in BBS management is to develop an efficient and durable treatment strategy that eliminates a patient’s need for an indwelling biliary device and the complications that come with it.

Over the last decade, the availability of single-use, low-profile cholangioscopes has led to a reemergence of percutaneous cholangioscopy, resulting in a paradigm shift in the diagnosis and management of biliary pathology.^9^, ^10^, ^11^, ^12^ Percutaneous transhepatic cholangioscopy (PTCS)–assisted laser stricturotomy has recently emerged as a promising new treatment for BBS. Unlike serial dilation protocols that rely on passive mechanical stretching to gradually remodel the narrowed duct, the laser addresses the pathology immediately by incising or debulking hyperplastic scar tissue. The holmium:yttrium-aluminum-garnet (Ho:YAG) laser is particularly well suited for this task due to its short penetration depth and lack of dispersion.^13^ Furthermore, the Ho:YAG laser can be utilized simultaneously for lithotripsy of biliary stones and cauterization of hemobilia, making it an attractive tool for endobiliary applications in the interventional suite.

The Percutaneous Endoscopic Benign Biliary Laser (PEBBL) study aims to prospectively evaluate the safety and efficacy of PTCS-assisted holmium laser stricturotomy over a 12-month period. The impetus for PEBBL comes from a small but growing body of evidence supporting the viability of this novel intervention as an efficient and durable solution to resolving benign biliary strictures and stones.^14^, ^15^, ^16^, ^17^ This pilot trial seeks to establish preliminary safety and efficacy benchmarks of this increasingly adopted therapy, and to serve as a framework for future investigations to build upon with the eventual goal of developing evidence-based guidelines for optimal patient selection, treatment protocols, and clinical outcomes.

## Materials and Methods

### Study Design

Institutional review board approval was obtained for this prospective, single-arm, open-label pilot trial conducted at a single academic institution (IRB #21–001910; National Clinical Trial #NCT05567003). All authors had access to the study data and reviewed and approved the final manuscript. Five patients with benign biliary strictures who met the eligibility criteria (Table 1) were consecutively enrolled in the PEBBL study between October 2022 and August 2023. Written and verbal informed consent to undergo PTCS-assisted laser stricturotomy was obtained. Interventions were performed by 3 board-certified interventional radiologists with 2–10 years of experience in percutaneous cholangioscopy. Baseline and follow-up data were collected at 3, 6, and 12 months and included routine magnetic resonance cholangiopancreatography (MRCP) or contrast-enhanced computed tomography (CT) imaging, clinic visits, and laboratory results including complete blood count, complete metabolic panel, and gamma-glutamyl transpeptidase. A grace period of ± 2 months was allowed at each follow-up interval to allow for minor variability in patient scheduling and adherence.

**Table 1:** Eligibility Criteria for PEBBL Enrollment

Enrollment eligibility
Inclusion Criteria
• Known benign biliary stricture
• Clinical evidence of biliary obstruction (current or prior)
• Age ≥18 y old
Exclusion Criteria
• Diagnosis of malignant biliary stricture
• Active cholangitis or sepsis at the time of index intervention
• Emergent indication for biliary decompression
• Liver transplantation within 90 d of enrollment
• Diagnosis of primary sclerosing cholangitis with ≥3 strictures
• Life-expectancy <36 mo

### Primary and Secondary Endpoints

The primary safety endpoint was a composite of all 30-day procedure-related adverse events, and the primary efficacy endpoint was technical success, defined as complete stricture resolution during the index intervention. The secondary safety endpoint was a composite of all 12-month procedure- and stricture-related adverse events. Secondary efficacy endpoints included primary patency, defined as freedom from stricture recurrence (determined clinically and/or radiographically), and cumulative device-free survival, defined as total time without a biliary drain or internal stent in place during the follow-up period (with the day of post-PTCS tube removal denoting time 0). Additional endpoints included secondary patency (freedom from stricture recurrence following a single reintervention) and percutaneous transhepatic biliary drainage (PTBD) tube-free survival (total time spent without a biliary drain following initial post-PTCS tube removal). Adverse events were reported according to the Society of Interventional Radiology Adverse Event classification system.^18^

### Devices

Percutaneous cholangioscopy was performed using the single-use SpyGlass Discover Digital Catheter system (Boston Scientific Corporation; Marlborough, MA, USA). The system includes a 10.8 French (3.6 mm) endoscope with a 65 cm working distance, equipped with a 3.6 French (1.2 mm) working channel, wide-angle camera, 120-degree 4-way tip deflection, dual irrigation channels with an aspiration port, and dual light sources.

Laser stricturotomy and lithotripsy (when applicable) were performed using the 300 μm diameter AccuTrac single-use laser fiber (Boston Scientific Corporation) equipped with a Holmium:yttrium-aluminum-garnet (Ho:YAG) laser. The Ho:YAG laser emits a wavelength of 2100 nm that is preferentially absorbed by water, resulting in a penetration depth of less than 0.5 mm in vivo with essentially no forward scatter or dispersion. Standard endovascular balloon catheters of various sizes and pressure ratings were used for balloon cholangioplasty, and standard multiside hole PTBD pigtail catheters were used for temporary preserved percutaneous access in the immediate postprocedural period.

### Patient evaluation

**Figure 1:**
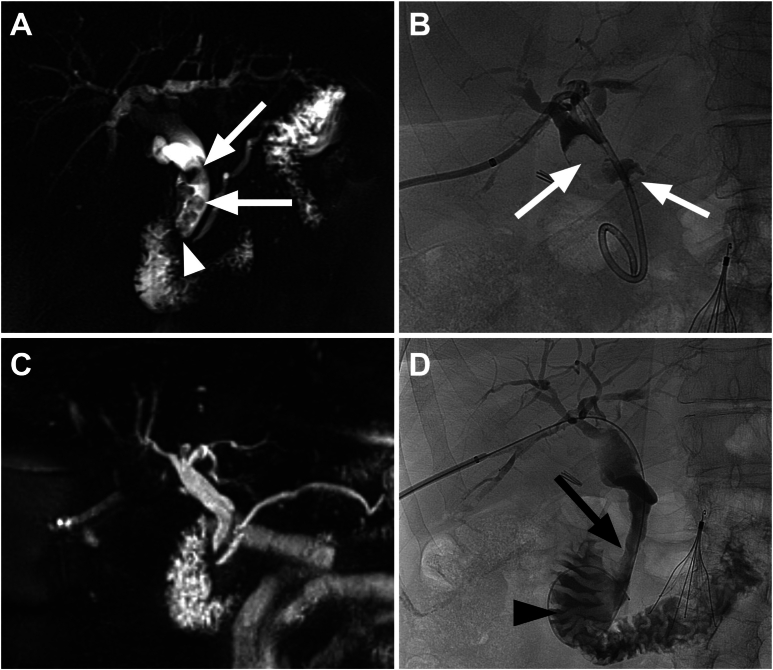
Case 1. Patient with a history of distal gastrectomy and biliroth II reconstruction 8 years prior, who subsequently developed choledocholithiasis and benign stricture of the distal common bile duct requiring internal–external percutaneous biliary drainage after failed ERCP cannulation of the ampulla. Preintervention MRCP (A) and cholangiography (B) demonstrate biliary dilation and multiple choledocholiths (white arrows) upstream to a tight focal stricture (white arrowhead). Laser lithotripsy and stricturotomy were performed. Follow-up MRCP (C) and tube-removal cholangiogram (D) show stricture resolution (black arrow) with with brisk antegrade bilioenteric flow (black arrowhead) through a widely patent distal CBD and no residual stones.

Patients underwent initial screening and baseline evaluation within 4 weeks of the index intervention. Patients who met the eligibility criteria and gave informed written and verbal consent to participate in the PEBBL trial were enrolled. All risks, benefits, and alternatives to the procedure were thoroughly discussed, and patients were allowed to exit the study at any point. The operating interventional radiologist conducted a clinical history and physical examination. Baseline data collected included patient demographics, medical and surgical history, and biliary stricture etiology and features. Hepatobiliary imaging was reviewed for procedural planning and documentation of stricture features. At a minimum, a baseline abdominal CT with intravenous contrast or magnetic resonance cholangiopancreatography (MRCP) study was required (Figure 1A). The degree of stenosis was calculated using MRCP or contrast-enhanced CT, with measurements based on the ratio of the stenotic segment diameter to the adjacent normal bile duct. Preprocedural laboratory studies included a basic metabolic panel, complete blood count, coagulation markers, and liver function enzymes. An international normalized ratio ≤ 1.5 and platelet count ≥ 50,000/μL was required before proceeding with PTCS. Any significant electrolyte abnormalities were corrected prior to intervention.

### Intervention

Procedures were performed in the outpatient or inpatient setting under general endotracheal anesthesia or monitored anesthesia care depending on patient comorbidities, cardiopulmonary risk factors, and Eastern Cooperative Oncology Group performance status. Prophylactic intravenous antibiotics with gram-negative coverage were administered within 1 hour of the intervention.

#### Percutaneous Cholangioscopy

An initial fluoroscopic cholangiogram was performed through a pre-existing transhepatic tract (Figure 1B). The tract was serially dilated to accommodate a 12 French peel-away sheath, and 2 0.035-inch Amplatz Super Stiff (Boston Scientific) guidewires (1 working wire and 1 safety wire) were advanced into the biliary tree. A peel-away rather than valved introducer sheath is preferred to prevent pressurization of the biliary system during irrigation with the cholangioscope. Over the working wire, the endoscope was advanced. The stricture morphology and surrounding biliary epithelium were visually inspected for mucosal lesions, edema, vascularity, erosions, synechiae, retained foreign bodies (eg, suture material), or biliary calculi. If a visible central ostium/concavity was present at a site of complete obstruction, initial recanalization was first attempted by gently probing with a 0.018” guidewire.

#### Laser Stricturotomy and Lithotripsy

**Figure 2:**
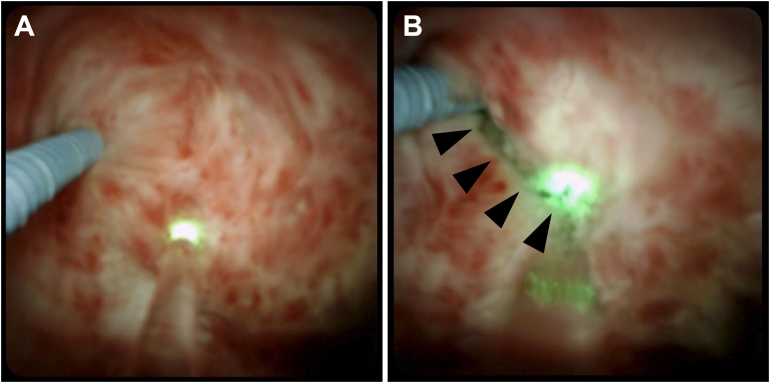
Case 2. Patient status post–orthotopic liver transplantation with duct-to-duct biliary anastomosis complicated by hepatic artery stenosis and biliary stricture. Recanalization attempts via ERCP and percutaneous transhepatic cholangiography were unsuccessful, requiring placement of an external biliary drainage catheter. Spyglass endoscopic images taken immediately before (A) and after (B) PTCS-guided laser stricturotomy. The operator has made a shallow 2 mm radial incision (arrowheads) through densely striated fibrotic bands of white tissue centered at the nidus of biliary obstruction.

**Figure 3:**
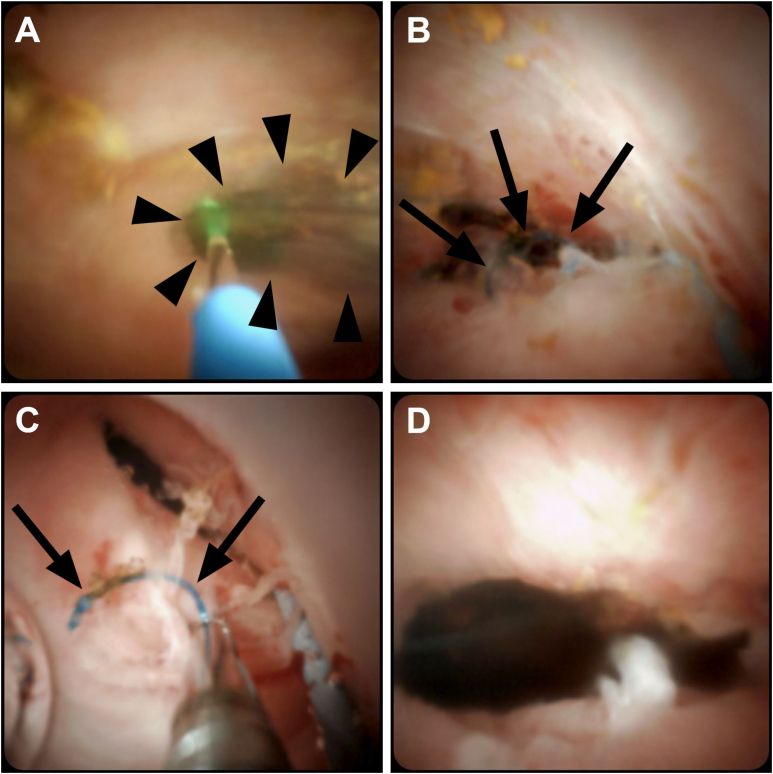
Case 4. Patient status post–orthotopic liver transplantation and Roux-en-Y hepaticojejunostomy complicated by biliary anastomotic stricture, recurrent cholangitis, and choledocholithiasis. After failed ERCP and 2 months of percutaneous biliary drainage, the patient underwent PTCS-guided laser stricturotomy and lithotripsy. Initial laser incision (A, B) through a thin membrane-like stricture (arrowheads) was performed, revealing a blue curvilinear foreign body consistent with suture material (arrows). Using the laser, the suture was released atraumatically and extracted with with endobiliary forceps (C). Continued laser debridement was performed until complete stricture resolution was achieved (D).

The Ho:YAG laser fiber was then passed through the endoscope working channel and advanced to the central most aspect of the stricture, marked by an area of abrupt tapering with characteristic white fibrotic tissue (Figure 2). If hepatolithiasis was present, lithotripsy was performed to fragment (1.0–2.0 J, 10–20 Hz) or pulverize (0.4–0.6 J, 30–40 Hz) the stones until all particulate matter and debris was cleared from the bile ducts. A dedicated retrieval basket or mini forceps were used to remove any stone fragment or exposed suture material amenable to retrograde extraction (Figure 3).

Laser stricturotomy was performed in short, less than 5-second bursts, cutting at a depth of no more than 1 mm of tissue at a time using laser energy settings of 1.0 J and 10 Hz. The technique varied by the nature of the tissue present at the stricture. Linear incisions were made for thin membrane-like tissue (Figure 3A) and fibrotic bands or synechiae when the distal course of the native bile duct was readily apparent. A spiral, corkscrew-like incision was made for longer strictures composed of dense fibrotic tissue, beginning centrally within the duct and advancing radially in centrifugal fashion until a 3–5 mm diameter lumen was created (Figure 2B).

Following stricturotomy and clearance of all debris, a final endoscopic and digital subtraction cholangiogram was performed to assess bilioenteric patency. A 10–12 French external or internal-external PTBD catheter was placed as a temporary safety access. Patients were discharged home the same day or transferred back to their inpatient unit after 4 hours of observation in the postprocedural recovery unit.

### Follow-up

Patients received a 10-day course of oral amoxicillin-clavulanate 875 mg twice daily. Ursodeoxycholic acid 300 mg twice daily was prescribed in patients with concomitant stone disease. Patients with a biliary catheter returned approximately 2 weeks after the index procedure for repeat cholangiography and tube removal after confirmation of adequate antegrade bilioenteric flow (Figure 1D).

Follow-up clinic or telehealth visits were scheduled at 3-, 6-, and 12 months postprocedure. Each follow-up visit included a history and physical examination, serum laboratory studies, and MRCP (Figure 1C). Patients with clinical, laboratory, or radiographic evidence of biliary obstruction (eg, new-onset biliary dilation, right upper quadrant abdominal pain, jaundice, cholangitis, or elevated bilirubin, alkaline phosphatase, or liver transaminases) at any point during the study period were considered to have stricture recurrence and underwent appropriate interventions. The study concluded at the end of the 12-month follow-up period.

### Data Analysis

Clinical, laboratory, and radiographical data were collected prospectively and analyzed by a statistician. Summary statistics and data are reported for each patient in the case series or expressed as the mean with standard deviation for continuous variables and frequency or number for categorical variables. Variables dependent on a time domain were reported as separate values for each time point. If applicable, univariate statistical comparisons were analyzed with the Wilcoxon rank sum test for continuous variables and the Fisher exact test for categorical variables.

## Results

### Study Population and Procedural Metrics

Five patients with BBS were enrolled in the PEBBL pilot trial. Patient demographics are listed in Table 2. The mean patient age was 55.8 years (24–79 years), and 4 (80%) of the patients were female. A majority of the cohort (4 of 5, 80%) had a history of biliary reconstruction complicated by anastomotic stricture, including 3 patients who were postorthotopic liver transplantation and 1 patient postcholecystectomy. Four patients (80%) had at least 1 prior failed attempt ERCP, and 4 patients (80%) had at least 1 prior hospitalization for cholangitis. The average degree of stenosis was 88.0% ± 13.0%; 2 patients presented with complete biliary obstruction at the time of PTCS. The mean cumulative time spent with an indwelling biliary catheter prior to the index procedure was 5.1 ± 5.6 months (range, 5 days–11.3 months).

**Table 2:** Patient Demographics and Procedural Data

Patient demographics	
Age (y)	55.8 ± 23.9
Sex (female)	4 (80%)
BMI (kg/m^2^)	27.9 ± 7.6
Past medical history	
Cirrhosis	2 (40%)
Cholangitis	4 (80%)
Pancreatitis	0 (0%)
Major gastrointestinal bleed	3 (60%)
Cardiovascular disease	2 (40%)
Diabetes mellitus	1 (20%)
Chronic kidney disease	2 (40%)
Malignancy^a^	1 (20%)
Immunocompromised state	3 (60%)
Past surgical history	
Biliary reconstruction (any)	4 (80%)
Orthotopic liver transplantation	3 (60%)
Roux-en-Y hepaticojejunostomy	3 (60%)
Billroth II gastrojejunostomy	1 (20%)

Values are presented as mean ± standard deviation or number (%).

a Fibrolamellar hepatocellular carcinoma status post orthotopic liver transplantation.

All patients underwent a single PTCS session. Procedures were performed under general endotracheal anesthesia (3 of 5) or monitored anesthesia care (2 of 5) in the inpatient (2 of 5) or outpatient (3 of 5) setting. Four patients had an existing PTBD catheter with a mature tract at the time of index intervention, while the fifth patient underwent a single-session intervention. All patients underwent adjunctive balloon cholangioplasty after PTCS-assisted laser stricturotomy, with balloons ranging from 6–12 mm in diameter. Other adjunctive PTCS-guided procedures included laser lithotripsy of biliary stones (2 of 5), anastomotic suture removal (2 of 5), and intrabiliary corticosteroid injection (1 of 5). A 10–12-French temporary internal-external (4 of 5) or external (1 of 5) PTBD catheter was left in place upon completion of the index PTCS intervention. Empiric antegrade balloon sweeping was performed in 1 patient at the time of catheter removal due to residual intraductal debris seen on cholangiography. Procedural history and BBS characteristics are outlined in Table 3.

**Table 3:** Biliary Stricture History, Features, and Procedural Data

Baseline BBS data	
Stricture etiology	
Anastomotic stricture	4 (80%)
Ischemic insult	2 (40%)
Allograft rejection	1 (20%)
Unspecified^a^	1 (20%)
Stricture features	
Complete obstruction	2 (40%)
Partial obstruction	3 (60%)
Degree of stenosis (%)	88.0 ± 13.0
Bismuth class 1	4 (80%)
Bismuth class 2	1 (20%)
Biliary stones present	2 (40%)
Time from stricture to PTCS (mo)	62.7 ± 84.7
History of failed ERCP	4 (80%)
History of failed BBS serial dilation	3 (60%)
Indwelling PTBD at time of PTCS	4 (80%)
PTBD duration pre-PTCS (mo)	5.1 ± 5.6
Procedural data	
Level of sedation	
General endotracheal anesthesia	3 (60%)
Monitored anesthesia care	2 (40%)
Procedure setting	
Outpatient	3 (60%)
Inpatient	2 (40%)
Adjunctive procedures with PTCS	
Laser stricturotomy	5 (100%)
Balloon cholangioplasty	5 (100%)
Lithotripsy of stones	2 (40%)
Anastomotic suture removal	2 (40%)
Intrabiliary steroid injection	1 (20%)
Radiation exposure	
Fluoroscopy time (min)	23.0 ± 10.9
Air kerma (mGy)	193.2 ± 147.0
Kerma area product (Gy^a^m^2^)	6.97 ± 15.6
Temporary biliary drainage catheter	
10 French	4 (80%)
12 French	1 (20%)
Internal-external PTBD	4 (80%)
External PTBD	1 (20%)
Time to drain removal (d)	22 ± 8.4
Estimated blood loss (mL)	<10 (100%)

Values are presented as mean ± standard deviation or number (%).

PTBD, percutaneous transhepatic biliary drain.

a Billroth II, anatomy with unclear stricture etiology.

### Efficacy Outcomes

Technical success was 100%, with each patient meeting the primary efficacy endpoint of stricture resolution at the index procedure (Table 4). Primary biliary patency at 12 months was 80.0% (4 of 5) and secondary patency was 100% (5 of 5). Temporary PTBD tubes were successfully removed in all 5 patients at a mean time to removal of 22 ± 8.4 days (range, 11–29 days) post-PTCS, and all remained tube-free through the study duration. The cumulative biliary device-free survival and PTBD tube-free survival was 9.4 ± 4.8 months and 11.6 ± 0.9 months, respectively. There was one case of asymptomatic stricture recurrence in a patient status post orthotopic liver transplantation complicated by graft rejection, hepatic artery stenosis, ischemic cholangiopathy, and choledocho-choledochal anastomotic stricture, who presented with a rising serum bilirubin 35 days after PTBD tube removal. This patient subsequently underwent ERCP and covered self-expandable metal stent placement after having previously failed both ERCP and percutaneous recanalization attempts prior to PTCS. Importantly, the patient did not require a repeat percutaneous intervention and remained PTBD tube-free through follow-up.

**Table 4:** Safety and Efficacy Outcomes at 12 Mo

Safety and efficacy data	
Patency outcomes	
Technical success	5 (100%)
Primary patency	4 (80%)
Secondary patency^a^	5 (100%)
Cumulative biliary device-free survival (mo)	9.4 ± 4.8
PTBD tube-free survival (mo)	11.6 ± 0.9
Early adverse events (≤30 d)	
Self-limited hemobilia (SIR class 1, mild)^b^	1 (20%)
PTCS tract site infection (SIR class 2, moderate)^b^	1 (20%)
Self-limited bile leak, fever (SIR class 2, moderate)^c^	1 (20%)
Late adverse events (>30 d, up to 12 mo)	
Recurrent stricture (SIR class 2, moderate)^c^	1 (20%)
Target lesion Reinterventions	
None	4 (80%)
ERCP/Metallic stent placement	1 (20%)
Radiographic patency (MRCP or CT)^d^	
3 mo	100 ± 0.0%
6 mo	100 ± 0.0%
12 mo	100 ± 0.0%

Values are presented as mean.

± Standard deviation or number (%).

a Defined as freedom from stricture recurrence with single reintervention.

b Same patient.

c Same patient.

d Excludes 3 patients at 3 mo and 1 patient at 6 mo who missed their scheduled imaging appointments.

Radiographic patency assessed by MRCP or contrast-enhanced CT at 3, 6, and 12 months was 100% (5 of 5) at each time point, and was supported clinically by the absence of symptoms of biliary obstruction and normalization of cholestatic serum markers. Serum laboratory results are listed in Table A1. Alkaline phosphatase levels (IU/L) decreased from a mean of 380.4 preintervention to 181.8, 148.5, and 130.0 at 3, 6, and 12 months, respectively. Mean total bilirubin levels (mg/dL) decreased from a mean of 0.82 preintervention to 0.68, 0.68, and 0.58 at 3, 6, and 12 months, respectively.

### Safety Outcomes

Three 30-day adverse events occurred in 2 patients related to PTCS, including 2 Society of Interventional Radiology (SIR) class 2 (moderate) and one SIR class 1 (mild) adverse events (Table 4). One patient had a self-limited episode of hemobilia (SIR class I) during a PTBD capping trial, followed by percutaneous tract site infection requiring oral antibiotics (SIR class 2) shortly after tube removal. This was the only patient in the cohort in whom the initial percutaneous biliary access and PTCS intervention was performed in a single session. The other 30-day adverse event was a self-limited perianastomostic bile leak identified during PTCS that resulted in fever requiring intravenous antibiotics (SIR class 2). It is unclear whether PTCS was the sole cause of this complication, as the patient had undergone 2 unsuccessful recanalization attempts (ERCP and percutaneous) within the same week and had intermittent fevers in the days leading up to PTCS.

Adverse events beyond 30 days included only the 1 patient with asymptomatic stricture recurrence who underwent covered self-expandable metal stent placement as previously discussed (SIR class 2). No severe complications or procedure-related deaths (SIR class 3–5) occurred during the study period. One patient died at 10 months follow-up due to unrelated complications of recurrent extrahepatic metastatic fibrolamellar hepatocellular carcinoma and remained otherwise tube-free until their demise without hepaticojejunostomy stricture recurrence.

## Discussion

The management of BBS remains a significant clinical challenge, particularly in patients who have failed conventional endoscopic or percutaneous therapies. While traditional approaches such as serial balloon dilation, silastic or metallic stent placement, and endobiliary radiofrequency ablation (ERFA) have demonstrated utility, their limitations—including high recurrence rates, prolonged catheter or other indwelling device dependency, and risk of deep tissue injury—underscore the need for safer and more durable alternatives.^19^, ^20^, ^21^ The Ho:YAG laser, especially when delivered percutaneously under direct cholangioscopic guidance, has emerged as a promising solution to refractory strictures, offering the ability of precise tissue incision with minimal collateral damage. PEBBL is among the first prospective studies to explore the feasibility, safety, and efficacy of percutaneous transhepatic cholangioscopy-assisted laser stricturotomy for the management of BBS, demonstrating promising preliminary safety and efficacy outcomes in a pilot cohort.

All 5 patients enrolled in PEBBL achieved technical success with immediate stricture resolution and durable biliary drain removal following a single PTCS session, with no severe adverse events. Four out of 5 patients remained stricture-free and device-free at 12 months, and all 5 patients were rendered biliary tube-free (and remained tube-free) throughout follow-up. One patient experienced asymptomatic stricture recurrence, which occurred in the context of hepatic artery stenosis and ischemic cholangiopathy following liver transplantation—factors known to independently predispose to recurrent biliary complications.^22^^,^^23^ Notably, this patient who had previously failed multiple interventions remained tube-free after salvage ERCP and metallic stenting, suggesting that PTCS-assisted holmium laser therapy can facilitate subsequent interventions in the face of complex anatomy or particularly challenging strictures.

Compared to other ablative technologies, the Ho:YAG laser offers unique advantages in tissue selectivity and safety. With percutaneous ERFA, stricturotomy is performed using a radiofrequency wire to thermally ablate fibrous scar tissue. Akinci et al. and Ozdemir et al. reported successful ERFA for refractory benign strictures in a series of 6 and 18 patients, respectively.^20^^,^^21^ However, a high incidence of thermal injury to the bile ducts and adjacent vasculature has been cited in the malignant biliary stricture literature, raising concerns for its application in benign strictures, especially given nonsterility of the biliary environment.^24^ A major advantage of the Ho:YAG laser is its pulsed monochrome wavelength of 2100 nm, which is preferentially absorbed by water resulting in a penetration depth of less than 0.5 mm *in vivo*.^25^, ^26^, ^27^, ^28^ Since tissues are composed mainly of water, all the holmium energy is absorbed superficially, thereby minimizing iatrogenic injury and inflammation to the deeper tissues.^13^^,^^20^ Moreover, ERFA lacks the same degree of spatial precision as the Ho:YAG laser, which enables short, controlled bursts of energy with submillimeter-level accuracy.^25^ These theoretical safety advantages were reflected in the PEBBL trial, where all patients underwent successful PTCS-assisted holmium laser stricturotomy without significant bile duct injury, hemorrhage, or other device-related major complication. The ability to deliver precise incisions under direct cholangioscopic visualization is especially critical in patients with complete biliary occlusion or surgically altered anatomy, where the native intraductal course is not as readily apparent during recanalization, thus underscoring the Ho:YAG laser’s utility in complex benign biliary strictures.

Akin to ERFA, high-frequency needle-knife electrotomy is another ablative modality that has been used as a tool with PTCS to treat benign biliary strictures. In 2021, Tao et al. published a series of 14 patients with post–liver transplant anastomotic strictures who underwent PTCS-assisted needle-knife electrotomy.^29^ They reported successful stricture resolution in 13 of 14 patients (92.9%) with a mean catheter-free follow-up duration of 15.7 months, although the mean catheter dwell time after stricturotomy was 7.1 months. There were 2 cases of cholangitis, 1 case of delayed hemobilia, and 1 stone recurrence without stricture recurrence. In comparison, the PEBBL trial achieved 100% stricture resolution with a mean time to catheter removal of 22 days, with one self-limited episode of hemobilia and bile leak and one tract site infection, with no severe bile duct injury, bleeding complication, or cholangitis. While both techniques appear to have high rates of stricture resolution, PTCS-assisted Ho:YAG laser stricturotomy offers significantly shorter catheter dwell times, in the range of days rather than months, which may support the notion that laser stricturotomy is superior at preventing thermal injury-induced inflammation, fibroproliferation, and unfavorable ductal remodeling.

Emerging retrospective data from PTCS-assisted holmium laser stricturotomy case series further support the findings in PEBBL. Lou et al. published a retrospective series of 15 patients who underwent PTCS-assisted holmium laser stricturotomy and lithotripsy for refractory postsurgical BBSs and hepatolithiasis, reporting 100% technical success with no major perioperative complications or bile duct injury at laser settings of 0.5–1.0 J and 6–10 Hz (ie, 3–10 W).^15^ The authors also introduced the “spiral” incision technique, which was adopted in the PEBBL trial for longer strictures composed of more dense, fibrotic tissue to create a 3–5 mm diameter neo-lumen. In 2022, Kord et al. reported a series of 4 patients with severe benign hepaticojejunostomy stenosis treated with PTCS-assisted holmium laser incision using 1.0 J at 10 Hz (10 W), achieving a median drain removal time of 14 days and sustained tube-free status over 1–9 months.^16^ Similarly, in 2023, Trivedi et al. described 5 patients with refractory benign post–liver transplant anastomotic strictures treated via PTCS-assisted stricturotomy using either the holmium or thulium laser.^17^ All strictures were successfully treated, with 18–22 French biliary drains removed after an average of 38 days, and all remained tube-free at a mean follow-up of 22 months without stricture recurrence. This favorable risk profile, combined with high rates of stricture resolution and minimal postprocedural burden, underscores the potential of PTCS-assisted laser therapy as a viable and durable option for managing complex BBS.

### Limitations and Future Directions

The PEBBL study has several limitations, including its single-center, single-arm design with small sample size. Given the low prevalence of BBS in the general population, there is an inherent challenge to recruiting patients prospectively and in a timely manner suitable for contemporary clinical trial designs. First, the study enrolled only 5 patients, which significantly limits both the statistical power and the generalizability of its findings. Additionally, the majority of cases in this series involved biliary strictures following orthotopic liver transplantation, whereas most benign biliary strictures (BBS) in the general population are secondary to iatrogenic injury secondary to cholecystectomy-related bile duct injury. This difference in etiology further restricts the applicability of these results to broader clinical practice. There was also variability in the procedural approach, with 1 patient receiving adjunctive intrabiliary corticosteroid injection during PTCS-guided stricturotomy. This lack of standardization introduces potential confounding and may disproportionately affect observed patency rates. Finally, given that time to stricture recurrence can vary widely, the relatively short follow-up period limits the ability to assess the long-term durability of patency beyond 1 year; future studies should aim for follow-up intervals of at least 24–36 months.

Given these limitations, future research should emphasize standardized treatment protocols, multicenter collaboration, and extended follow-up. Although the rarity of BBS presents challenges to timely prospective enrollment, collaborative efforts such as clinical registries are essential to collecting real-world, generalizable data. Initiatives are already underway to incorporate PTCS interventions into the VIRTEX SIR Data Registry, with additional multi-institutional efforts expected to follow. Future studies should incorporate cost-effectiveness analyses, including direct procedural costs, hospital stay expenses, and long-term health-care utilization, to support clinical decision-making for PTCS-assisted holmium laser stricturotomy in benign biliary stricture management.

## Conclusion

PTCS-assisted holmium laser stricturotomy is a promising new therapy for benign biliary strictures that can expedite and sustain catheter or stent removal in tube-dependent patients with otherwise limited treatment options. This emerging treatment strategy has the potential to replace prolonged stenting protocols with a more immediate and more durable means to achieve a tube-free state. PEBBL was a single-arm prospective pilot trial demonstrating favorable safety and efficacy in a small cohort at 12 months. However, more data are needed at this time to reproduce and confirm these results at scale. This study should serve as a framework for future investigations and sets preliminary safety and efficacy benchmarks for others to build upon.
