# Supplementary material for: Twelve-month Results From the Percutaneous Endoscopic Benign Biliary Laser Stricturotomy Study: A Prospective Single-Arm Pilot Trial Evaluating Safety and Efficacy
Source: Gastro Hep Adv. 2025 Aug 25;5(1):100770. doi: 10.1016/j.gastha.2025.100770 (PMC12546874; doi:10.1016/j.gastha.2025.100770)
Supplement: Supplementary Table 1 [file mmc1.docx]

**Supplementary Table 1.** Serum laboratory values.

| **Laboratory Data** | | | | |
| --- | --- | --- | --- | --- |
|  | Pre-PTCS | Post-PTCS | | |
|  |  | 3-month | 6-month * | 12-month |
| Total Bilirubin (mg/dL) | 0.82 ± 0.8 | 0.68 ± 0.4 | 0.68 ± 0.5 | 0.58 ± 0.3 |
| Alkaline Phosphatase (IU/L) | 380.4 ± 270.0 | 181.8 ± 79.1 | 148.5 ± 55.6 | 130.0 ± 25.5 |
| Aspartate aminotransferase (IU/L) | 65.6 ± 37.9 | 72.4 ± 98.6 | 26.0 ± 6.1 | 118.0 ± 207.5 |
| Alanine aminotransferase (IU/L) | 64.8 ± 33.6 | 55.2 ± 77.3 | 19.5 ± 2.6 | 80.8 ± 127.0 |
| Total Protein (g/dL) | 6.8 ± 1.1 | 6.6 ± 0.6 | 7.0 ± 0.3 | 6.6 ± 0.7 |
| Albumin (g/dL) | 3.7 ± 0.5 | 3.8 ± 0.5 | 4.1 ± 0.3 | 3.9 ± 0.5 |
| White blood cell count (x10^9^/L) | 9.4 ± 6.0 | 6.6 ± 1.0 | 6.6 ± 1.1 | 7.9 ± 2.3 |
| Platelet count (x10^9^/L) | 440.2 ± 448.0 | 269.8 ± 145.1 | 222.8 ± 98.2 | 222.6 ± 47.3 |
| Creatinine (mg/dL) | 0.80 ± 0.2 | 0.85 ± 0.1 | 0.85 ± 0.1 | 0.86 ± 0.1 |
| Values are presented as mean ± standard deviation.  *6-month laboratory data missing for 1 patient. | | | | |
